# Peptides for Infectious Diseases: From Probe Design to Diagnostic Microarrays

**DOI:** 10.3390/antib8010023

**Published:** 2019-03-12

**Authors:** Marina Cretich, Alessandro Gori, Ilda D’Annessa, Marcella Chiari, Giorgio Colombo

**Affiliations:** 1 Consiglio Nazionale delle Ricerche, Istituto di Chimica del Riconoscimento Molecolare (ICRM), Via Mario Bianco 9, 20131 Milano, Italy; marina.cretich@icrm.cnr.it (M.C.); alessandro.gori@icrm.cnr.it (A.G.); ilda.dannessa@gmail.com (I.D.); marcella.chiari@icrm.cnr.it (M.C.); 2 Dipartimento di Chimica, Università di Pavia, V.le Taramelli 12, 27100 Pavia, Italy

**Keywords:** molecular dynamics, microarrays, diagnostics, computational chemistry, computational biology, peptides

## Abstract

Peptides and peptidomimetics have attracted revived interest regarding their applications in chemical biology over the last few years. Their chemical versatility, synthetic accessibility and the ease of storage and management compared to full proteins have made peptides particularly interesting in diagnostic applications, where they proved to efficiently recapitulate the molecular recognition properties of larger protein antigens, and were proven to be able to capture antibodies circulating in the plasma and serum of patients previously exposed to bacterial or viral infections. Here, we describe the development, integration and application of strategies for computational prediction and design, advanced chemical synthesis, and diagnostic deployment in multiplexed assays of peptide-based materials which are able to bind antibodies of diagnostic as well as therapeutic interest. By presenting successful applications of such an integrated strategy, we argue that they will have an ever-increasing role in both basic and clinical realms of research, where important advances can be expected in the next few years.

## 1. Introduction

Peptides are extremely versatile molecules with a wide range of applications. Peptides are attractive for chemists and chemical biologists thanks to the rich diversity of chemical functionalities they can potentially display. Indeed, amino acid building-blocks recapitulate all of the main traits of organic chemistry reactivities through their side chain groups, which range from hydrophobic and aromatic, to hydrophilic, acid, basic, and charged. The synthetic accessibility of linear chains through solid-phase methods, combined to the possibility to further derivatize side chains, opens access to a wide spectrum of chemical diversity and reactivity for the products. 

It comes as no surprise that the use of peptides and peptidomimetics in drug discovery in the development of peptide-based materials for medical devices, and even in vaccinology, has recently met with renewed enthusiasm. Indeed, peptides play key roles in a number of biochemical pathways in and outside cells, making the discovery of artificial sequences (or mimics) which are able to either interfere or synergize with the respective natural processes an attractive strategy towards the production of new materials: as an example, the advantages they can offer as drugs are specificity, potency, and low toxicity. However, practical hurdles such as their poor stability, short half-life, and the fact that they are targeted by proteases have somewhat limited the realization of their full potential in medicinal chemistry. 

While these limitations still call for significant efforts in the pharmacological setting, peptides and peptide-based derivatives can certainly offer great opportunities in the field of probe development for disease diagnostics.

In this framework, peptide microarrays are appealing tools as they allow the simultaneous screening of hundreds to thousands of different sequences (and potentially structures, if conformational control is introduced) immobilized in spatially ordered patterns of spots on solid supports [1,2]. After incubation with a biological sample, specific interactions that take place on the single spots are detected in a label-free mode by surface plasmon resonance or mass spectrometry or in a label-dependent manner through fluorescence, chemiluminescence or colorimetry. 

Peptide microarrays are increasingly being used in the biomedical area for the screening of enzyme function and inhibition [3] and to profile the immune-responses to pathologies by the capturing of disease-related antibodies [4,5] from body fluids. 

Despite great improvements in the time-to-result and the excellent sensitivity and specificity provided by nucleic acid-based approaches to test for infectious diseases (PCR, sequencing, and DNA microarrays) [6,7], serological methods have a number of advantages including their low cost, multiplexing capacity and ease of coupling to simple devices such as lateral flow tests for use with minimal or no laboratory infrastructures. 

In this context, it is now well appreciated that, in many disease conditions, antibody-mediated responses may precede the manifestation of actual symptoms, sometimes even for a long time. This holds true for bacterial and viral infections. If the immune system has responded to a threat with a B-cell response, it is reasonable to assume that circulating antibodies, elicited specifically against molecular targets on pathogenic factors, could aptly represent efficacious biomarkers of the disease. The capture and detection of such antibodies expectedly requires the availability of their respective antigens as probes. 

To this end, the use of whole (attenuated) pathogens poses relevant problems as micro-organisms may be hard to culture in vitro, and therefore their production is impractical. 

The advent of reverse vaccinology has given a huge boost to the discovery of tools that are more manageable and amenable to use as molecular probes. Reverse vaccinology applies bioinformatic methods to mine pathogen genomes to identify protein antigens (Ag) that are typically expressed on the outer membrane surface, and are thus most prone to be recognized by molecules of the immune system [8,9,10,11]. Typically, this is achieved by identifying specific sequence motifs, such as those encoding for outer membrane proteins. The predicted Ag is then expressed, produced, and used in subsequent experimental tests. Full-length proteins, however, may themselves be difficult to handle, suffer from stability problems and in some cases may be recognized by more than one single Ab, giving rise to potential misinterpretations in the diagnostic setting. 

A plausible way to overcome these obstacles would be to identify the regions of the antigen that are specifically recognized in the course of an immune response. Chemical and structural information on such regions, called epitopes, could then be used as template to develop mimics that bind specifically to the antigen-binding pocket of the desired disease-relevant antibody. Peptides and peptidomimetics represent the ideal chemical tools to realize small epitope mimics that recapitulate the essential determinants of immune-reactivity and Ab-recognition of the cognate full-length antigen.

At this point, two main problems need to be tackled. The first lies in the identification of the epitope region on an antigen, most often in the absence of detailed X-ray information on the relevant antigen–antibody complex. In this context, sequence and structure-based computational methods have played an increasingly relevant role in recent years, facilitating the identification of molecules which are able to efficiently engage disease biomarkers. 

The second concerns the optimization of the presentation of the synthetic immunoreactive molecule(s) on diagnostic devices to efficiently capture the desired Abs. Circulating antibodies produced against disease-specific antigens can in fact be detected by passing patient fluids (serum, plasma, etc.) over an immobilized bait and then measuring the amount of antibody retained with a labeled secondary antibody. Novel methods of oriented display, as well as the use of optimized surfaces for the attachment of the probes, can aptly provide a significant increase in the performances of novel diagnostic tools. 

From this perspective, we will present and discuss the work, views and methods we have developed and integrated in our laboratory to discover, design, synthesize, and display novel peptide-based molecules with the aim of realizing the potential of epitope-mimicry in the development of efficient diagnostic methods for the detection of disease biomarkers. 

## 2. Epitope Prediction

The initial condition to start the design of small peptide-based mimics of the immunoreactive regions of antigens depends on the capability to correctly locate the privileged substructures that are preferentially engaged in antibody binding. In this context, computational approaches have played a constantly increasing role over recent years. Sequence-based methods and algorithms based on the combination of descriptors reporting on solvent exposure, hydrophobicity profiles, and geometrical properties have provided important results [12,13,14,15,16,17,18,19,20,21]. 

However, it has become increasingly clear that a combination of factors spanning the sequence, structure, dynamics and location of specific protein regions determines the cross-talk between the antigen and the antibody. In principle, the identification of (surface-exposed or exported) antigen substructures that display peculiar sequence and dynamic properties is the basis for the design of peptidic interactors as probes for Ab detection. In this framework, epitope sequences tolerate mutations, suggesting that they are not involved in the stabilization of the antigen fold. These sites continuously evolve to escape recognition by the immune system of their host, without disrupting the global 3D fold required for the function of the protein they belong to, which would ultimately be detrimental for the survival of the pathogen. Furthermore, epitopes are in general flexible and support extensive conformational fluctuations which underlie the conformational selection mechanisms that underpin antibody binding. Finally, epitopes define large exposed patches on the protein surface. 

To put these considerations in a quantitative predictive model, we introduced MLCE (matrix of low coupling energies) [22,23]. The prediction is based on the energy decomposition method [24,25,26], which allows the detection of residue–residue couplings that are important in the stabilization of a fold (see details in the next paragraph). The method provides a simplified view of residue–residue pair interactions, extracting the major contributions to the energetic stability of the native structure from the results of all-atom molecular dynamics (MD) simulations. For a protein of *N* residues, the *N* × *N* matrix (*M_ij_*) of average nonbonded interactions between pairs of residues can be built by averaging over the structures visited during an MD trajectory. The resulting energy matrix is then simplified through eigenvalue decomposition. The analysis of the *N* components of the eigenvector associated with the lowest eigenvalue was shown to identify residues that behave as maximally strong or maximally weak interaction centers. The principal eigenvector can be used to reconstruct a simplified version of the energy matrix, which recapitulates the residue pairs with maximal and minimal couplings with the native structure of the protein. The simplified map is next filtered with topological information to identify patches of local couplings characterized by energetic interactions of minimal intensities. Low-intensity couplings between distant residues in the structure are a trivial consequence of the distance-dependence of energy functions. However, low-energy couplings between residues that are proximal in the folded 3D structure of the protein identify those sites which are particularly frustrated (non-optimized), which can therefore be regarded as the preferential perspective points of interaction with binding partners. Moreover, such regions would tolerate mutations without dramatic consequences on the antigen three-dimensional structure [22,25,26]. 

**Figure 1:**
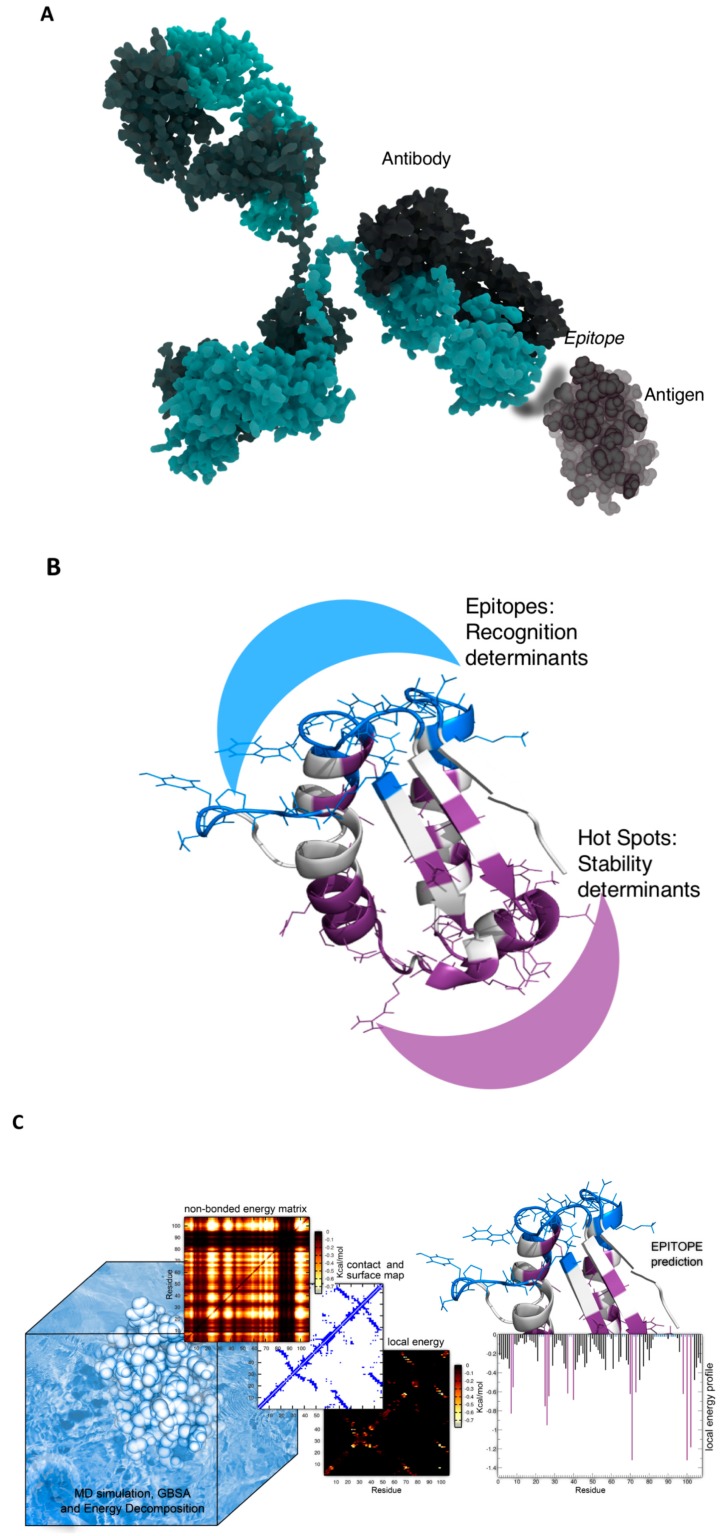

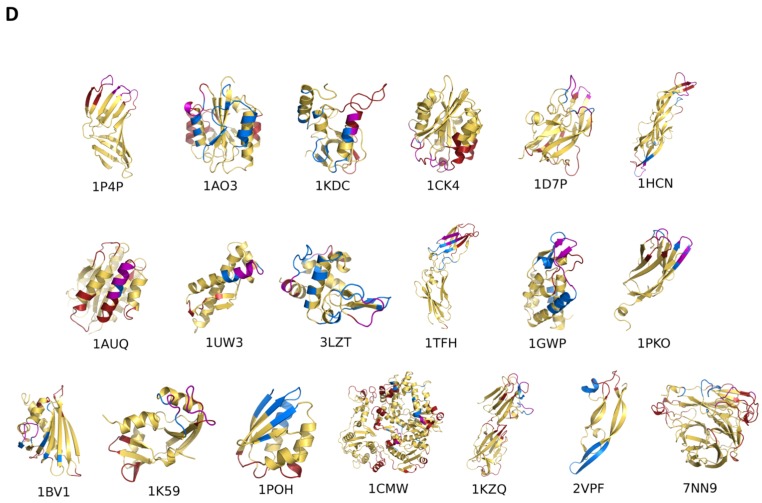
Structure and energy-based strategy for the prediction of antibody binding regions from protein antigen structures. (**A**) Definition of the epitope region on a protein antigen prone to engage an antibody; (**B**) energy-based separation of the folding core and interaction regions in antigens; (**C**) matrix of low coupling energies (MLCE) strategy; (**D**) epitope prediction and benchmark on a number of proteins extracted from the Protein Data Bank.

Because it is based on a preliminary characterization of the structural ensemble of the antigen by molecular dynamics (MD) simulations, the method naturally takes into account information on the conformational transitions that underlie the adaptation to the respective antibody to form a complex. Overall, MLCE showed the unique ability to provide information on the epitope sequence, structure and flexibility. A pictorial scheme of the method is reported in Figure 1. 

The performances of the method were first benchmarked on a set of proteins whose complexes had been solved by X-ray crystallography. We then extensively applied it to the prediction of initial candidate probes for the early diagnosis of bacterial and viral infections. 

Successful application was reported for the identification of new peptide-based diagnostic probes to detect *Burkholderia pseudomallei* infections. 

*Burkholderia pseudomallei* is the etiological agent of melioidosis, a pulmonary infection that is widely diffused in South East Asia and Northern Australia [27]. The bacterium is currently spreading to other areas of the world due to climate change and migration. Despite being associated with a mortality rate of more than 40%, no diagnostic tool to quickly and efficiently report on the infection caused by the bacterium is present on the market. 

In this context, we started from the knowledge of the 3D structure of a number of protein antigens and applied the MLCE approach to predict the sequence, structures and location of candidate epitopes [28,29,30,31,32,33,34]. Interestingly, the predicted sequences, once realized in the form of isolated peptides and displayed for testing with sera/plasma on ELISA plates, showed diagnostic performances similar to those of their cognate full-length proteins. 

In one particular case [30], an apparent discrepancy was noticed between the computational prediction and experimental epitope characterization based on tryptic digestion followed by antibody capture. Indeed, polyclonal antibodies may be induced by several factors in vivo, including degradation of the antigen into smaller fragments, which may ultimately lead to the presentation of sequences that are not on the surface or are inaccessible in the crystal structure. Such epitopes would not be predictable by MLCE. To overcome this limitation, MLCE was combined with a domain decomposition approach; the latter is an extension of the eigenvalue energetic analysis of the folded state and views a large protein as a combination of independent folding units [26]. In this context, the method uses combinations of non-redundant eigenvectors and filters out only specific subsets of strong interactions that are shown to correspond to independent folding nuclei. Grouping different proximal folding nuclei based on a proximity criterion permits the definition of structural domains. The use of the domain decomposition approach permitted us to identify possible boundaries with the antigens, which computationally mimicked a partial proteolysis. The application of the MLCE prediction to the resulting isolated domains identified new potential epitope sequences that turned out to display a high reactivity when tested against sera of *B.pseudomallei*-infected patients. 

The MLCE approach was subsequently applied to the problem of developing diagnostic probes for ZIKA virus (ZIKV) infections. We focused on ZIKV non-structural 1 (NS1). In this case, MLCE identified a putative antigenic region of NS1. In this case, the approach returned a region of the protein encompassing different secondary structure motifs. Once realized and combined for co-presentation to patient sera, several candidates were found to be effective in discriminating ZIKA-positive vs. healthy samples [35].

It is worth noting here that other computational and experimental approaches have been proposed and shown to perform with good success.

Fiorucci and Zacharias introduced the electrostatic desolvation penalty (EDP) method [21]. This approach uses probe molecules to evaluate the tendency for a certain surface region to be engaged in binding by another protein: the central idea of the method is that surface regions with a small free energy penalty for desolvation correspond to potential sites of interaction. Specifically, they calculate the electrostatic free energy penalty or gain in placing a neutral low-dielectric probe at various protein surface positions, using the finite-difference Poisson equation. Very interestingly, upon benchmarking against a large set of known general protein–protein interactions, the correct binding site overlapped with one of the six regions of the lowest electrostatic desolvation penalty in more than 90% of cases. The specific applications to antigen–antibody complexes demonstrated that EDP could be aptly extended to the difficult case of epitope prediction. This method requires no initial training, as the epitope propensity of a sequence is evaluated based on its physico-chemical properties. 

Ellipro represents another method that does not require training [14]. The working hypothesis at the basis of this method is that residues that protrude from the protein surface are more accessible for Ab binding [14]. The protein is treated in a simplified way as an ellipsoid and the Thornton’s method to identify protruding residues is combined with a residue-clustering algorithm to define the final predicted B-cell epitopes. 

The computer method SEPPA [15] makes use the concept of ‘unit patch of residue triangle’ to define the spatial context of residues in a protein surface, with the hypothesis that epitope regions should have specific compactness profiles on the surface. SEPPA gave an average AUC value over 0.742 and produced a successful pick-up rate of 96.64% when tested on validated databases. 

PEPITO combines propensity scales with surface exposure information [36,37] and utilizes the Discotope amino acid propensity scale and side-chain orientation as well as solvent accessibility data. 

Sela-Culang and coworkers introduced a mixed computational experimental method to B-cell epitope identification [38]. Using the sequence of an antibody, the authors proved it possible to identify discontinuous epitopes on the targeted antigen. The method uses residue-pairing preferences and additional interface information. Predictions are improved by combining them with data on cross-blocking experiments that identify groups of antibodies with overlapping epitopes. 

Other exquisitely experimental approaches to epitope identification/characterization involve mass spectrometry (MS) approaches based on hydrogen/deuterium exchange (HDX), phage display based methods, and high-throughput synthesis and analyses. 

HDX-MS for epitope discovery is generally coupled to limited proteolysis approaches in epitope mapping problems: specifically, an antigen is digested by an array of proteases in the presence or absence of its antibody. The fragmentation patterns resulting from proteolysis are compared [39,40,41]. The presence of the antibody bound to its epitope may protect the cleavage site at or near the epitope itself. This technique is generally fast, relatively inexpensive, and has been shown to be able to quickly identify regions of interaction in an antigen [31,42,43]. In some cases, HDX combined with limited proteolysis has been coupled to liquid chromatography (LC) [44,45]. 

Phage display represents another method to select linear and discontinuous epitopes. The latter can be selected using purified antibodies or preparations of polyclonal serum. In this approach, linear epitopes as well as structurally organized ones, constrained by disulphide bonds, can be displayed on very large phage libraries. Antibodies are subsequently used to pan the epitopes. The targets that get recognized can be sequenced to determine the sequences. 

Further popular strategies for epitope mapping consist of testing the binding of antibodies against multiple peptide sequences that are prepared with medium to high-throughput synthetic techniques. In some cases, overlapping sequences spanning the whole antigen sequence are tested for their antibody-binding capability; peptides found to bind are used to define an epitope. One potential drawback of this approach is that it may miss the recognition of discontinuous conformational epitopes. Based on these concepts, Kodadek and coworkers have shown impressive results in the identification and optimization of chemical tools to manipulate antibody recognition and capture pathogenic antibodies related to chronic lymphoid leukemia (CLL) or autoimmune disorders with high selectivity. To this end, they exploited a high-throughput screening protocol that used a large library of bead-displayed oligopeptides, generated via split-and-pool synthesis. This approach first concentrated on removing ligands, as the antigen-binding sites of antibodies that are not disease-related are first removed from the pool. Next, to identify ligands to disease-related antigen-binding sites, the remainder of the library was incubated with a soluble version of a patient-derived CLL. For an antibody in the presence of a large excess of competitor proteins, the antibody-binding beads were identified by the binding of a secondary antibody linked to a fluorescent probe. These active binding compounds were thus isolated and characterized with mass spectrometry. With this approach the authors are able to identify and further develop sequences or peptidomimetics that bind in the low nanomolar range. These approaches and their subsequent application in therapy and diagnostics are described at length in Doran et al. [46]

## 3. The Importance of Probe Presentation: Conformation and Surface Chemistry Matters 

The structure-based prediction of potentially reactive sequences is a necessary first step in any project aimed at developing new molecules with interesting applications. Complementary to this, the presentation/display of designed sequences in the best spatial orientation for immunoreactivity with patients’ samples represents a fundamental step in the quest for devices with optimal performances. 

**Figure 2:**
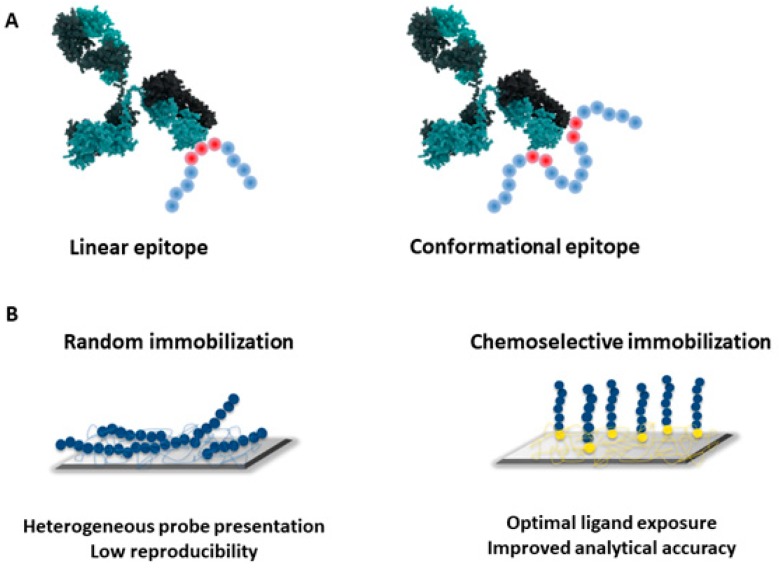
(**A**) Schematic illustration of linear vs. discontinuous epitopes; (**B**) random immobilization strategies may result in non-homogeneous probes presentation to the target antibodies, mining the reproducibility of the analytical data; conversely, chemoselective immobilization allows for the fine-tuning of probe exposure at the solid–liquid interface, enhancing the analytical performances.

Peptide microarrays are composed by a variable number of peptides either in situ synthesized step by step on a suitable flat support or printed after individual synthesis and purification. In situ methods, based on spot synthesis of peptides (SPOT), particle-based synthesis and photolytographic methods, generate high-density arrays of thousands of peptides, potentially providing a huge amount of information by the linear scanning of aminoacidic sequences. This approach, reviewed elsewhere [47], is especially useful when no or poor structure-based information is available; it is high-throughput and uses minimal amounts of reagents. On the other side, the purity and the quality of the synthesized probes cannot be assessed, and a consolidated expertise in data analysis is required to filter out and interpret the large amount of generated results. Moreover, this approach largely limits the amount of information related to the presence of discontinuous epitopes, i.e., immunoreactive regions generated by non-contiguous amino acid patches brought in close contact by the 3D-arrangement of the antigen structure, that represent most of the proteins’ actual epitopes (Figure 2A). However, short peptide sequences are rarely capable of assuming a 3D structure out of their native context and, in addition, a simple peptide surface physisorption or non-specific covalent binding are unfavorable immobilization methods due to the possible masking of key epitope residues. All together, these challenges can impair bioassay performance through ambiguous results and reduction in sensitivity. In this scenario, our workflow for the development of peptide microarrays is quite opposite, as it focusses on selected small epitope libraries, where their conformational dynamics and surface presentation are finely tuned. 

The conformational control of peptides is generally performed by stapling and cyclization techniques that are based on the formation of intramolecular covalent bonds or on the use of anchoring macrocyclic rigid scaffolds to thermodynamically favour a limited set of peptide conformations [48,49]. In our previous work, we critically assessed the role of epitope conformational stabilization in the development of immunoreactive probes using a triazole-mediated stapling strategy to stabilize the native α-helical fold of the Pal3 peptidic epitope from the *Burkholderia pseudomallei* protein antigen Pal_Bp_ [32]. We compared the diagnostic performances of the linear epitope (Pal3), which showed no propensity to fold outside its native protein context, with those of its stapled analogue Pal3H, which in contrast retained a strong helical propensity. Interestingly, Pal3H proved more effective in discriminating between melioidosis patient subclasses, highlighting the role of conformational stabilization in favoring an optimal bioprobe presentation to the target antibodies. 

In this direction, limiting heterogeneous bioprobe presentation onto analytical surfaces is crucial to guarantee consistent device performances and reliable diagnostic outcomes (Figure 2B).

**Figure 3:**
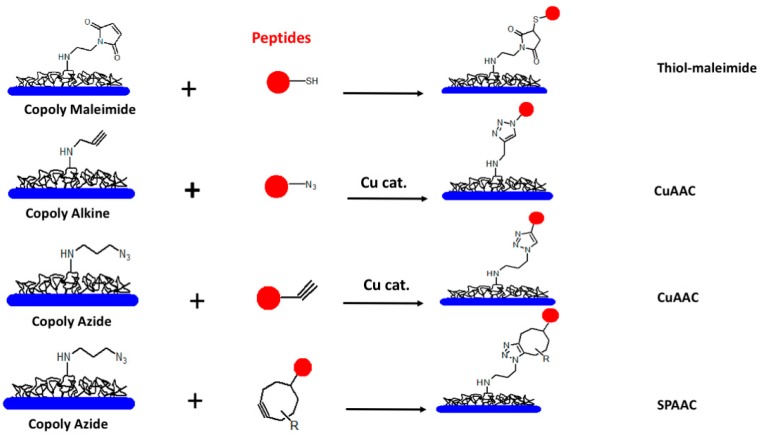
Family of clickable polymeric coatings generated from the common precursor Copoly (DMA-NAS-MAPS) to generate a polymeric platform for chemoselective peptide binding on microarrays. Modified peptides react by sulfhydryl-maleimide conjugation on Copoly maleimide; copper catalyzed azide-alkyne-cycloaddition (CuAAC) on Copoly alkine and Copoly azide; copper-free strain-promoted-azide-alkyne-cycloaddition (SPAAC) on Copoly Azide.

We have deeply investigated this aspect and developed new chemical strategies to gain optimal epitope display in our peptide-based bioassays. Our approach for surface modification is based on the use of polymeric coatings that rapidly self-adsorb on the analytical surface from a diluted water solution, forming an anti-fouling and hydrophilic layer bearing functional groups for the covalent immobilization of bio-probes [50,51]. Besides these polymeric coatings, able to non-selectively react with any nucleophiles [52], we have recently introduced a family of “clickable” polymers combining the properties and ease of coating of the parent polymer with chemo-selective reactivity towards orthogonal groups in the binding probes [53] (Figure 3).

Click chemistry reactions in fact have exquisite chemoselectivity towards common functional groups that can be easily introduced into peptides providing a high-yielding and cost-effective immobilization strategy. Moreover, benign reaction conditions and fast reaction kinetics that commonly characterize this class of reactions address the need for fast immobilization well, which is particularly relevant to overcoming the intrinsically slow reactivity at the solution–solid support interface [54]. 

A number of click-type immobilization strategies were compared in the frame of the peptide-based sero-diagnostics of *Burkholderia* infections in cystic fibrosis patients [55]. In this work, we showed that not only the random immobilization of peptides is not feasible for a reliable diagnosis in complex samples, but also that the probe binding and reproducibility of the assay are poorly consistent when this approach is used. In contrast, peptide orientation via chemoselective immobilization was successful in enhancing the statistical significance of the test, further improved when the peptides were spaced and better exposed on the chip surface by means of small polyethylene glycol (PEG) linkers. Overall, all the click chemoselective reactions that we evaluated (thiol-maleimide and azide-alkyne) showed a comparable efficiency in terms of diagnostic performance. The binding efficiency to microarray surfaces was the highest when using azide-alkyne-cycloaddition (both in the Cu-catalyzed and Cu-free versions); on the other hand, the best spot morphology and fluorescence were obtained by using thiol-maleimide conjugation. This approach, however, is not general and is only applicable when cysteine residues are not present in the selected peptide sequences. 

Our results highlighted the challenges of real-case serological assays, where the in vivo response to immunogenic epitopes derived from infections is associated to a complex antibody repertoire that targets several conformers of the same epitope. In this type of assays, the polyclonality of patient’s response enhances non-specific interactions while the probe discrimination potential relies on a narrow border between signal detection and background noise. In this context, to gain optimal specificity and sensitivity, the whole dynamic distribution of peptide epitope conformers must be fully accessible to the range of antigen-directed elicited antibodies. To this end, any strategy targeted at enhancing the affinity of the immobilized probes for disease-specific antibodies, including space-oriented display of epitopes, fine control of probe density and multivalent presentations, is beneficial to the delivery of robust clinical tests [56]. 

**Figure 4:**
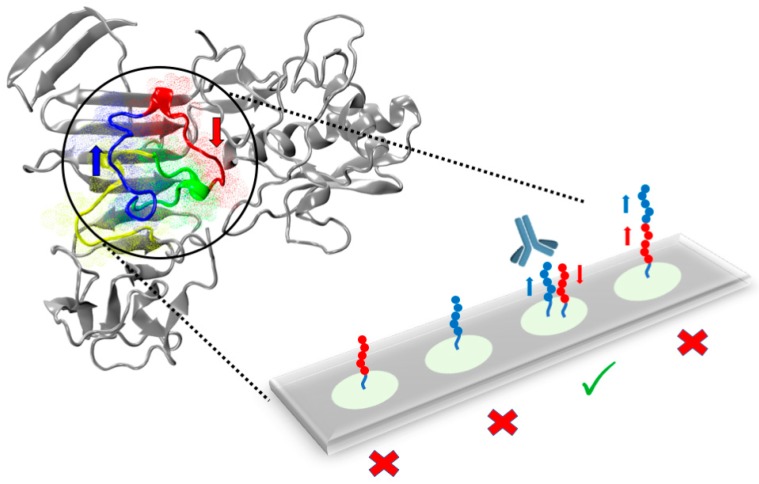
Strategy for the functional mimicking of discontinuous epitopes by the oriented and spatially controlled co-immobilization of proximal peptide sequences forming the putative antigenic region within the native antigen fold.

A fine-tuning of the probe density and orientation may be particularly useful to discriminate infections that present a high degree of antibody cross-reactivity, such as in the case of arboviruses. In this frame, the combined use of multiple peptides could provide superior discriminative capacity than a single probe by the generation of an immune-signature. In the case of Zika virus NS1 protein, we found that antigenic response is determined by discontinuous epitopes arising from spatial arrangements of non-contiguous linear peptide sequences. This could represent a severe limitation to the applicability of peptide microarrays in diagnostic tests. To address this limitation, starting from the computationally-guided identification of a surface antigenic NS1 region, we functionally mimicked the native antigen fold through the oriented and spatially controlled co-immobilization of proximal peptide sequences forming the putative antigenic region (Figure 4). 

This straightforward method enhanced the discriminative performance of single linear peptides, expanding the toolbox for immune-diagnostic peptides to the mimicking of antigenic surfaces [35].

As biomolecule 3D properties are so intimately linked to their analytical performances, a further and innovative step to preserve their native structure and function upon surface immobilization is to gently encapsulate them within hydrogel matrices under solution mimetic conditions. This strategy is associated with additional advantages including increased loading capacity, reduced nonspecific binding and an enhanced signal-to-noise ratio. Most of the reported hydrogels for microarrays are based on polymeric and biopolymeric matrices. However, it is often not trivial to match analyte diffusion rates through the gel matrix with stable bioprobe entrapment. In addition, laborious strategies may be required for hydrogel chip fabrication due to the viscosity of common hydrogels. We tackled these limitations by developing a nanostructured self-assembling peptide hydrogel (YF-Q11) characterized by favorable properties for 3D microarrays [57]. These include both low viscosity, enabling its direct use during automated spotting, combined with good mechanical robustness and, most relevantly, unimpaired and tunable diffusion properties of different macromolecules (peptides, proteins, antibodies). 

Overall, we obtained a stable and yet highly permeable semi-wet matrix to run ultrafast (<10 min) fluorescence immunoassays under solution-like conditions. Importantly, the hydrogel matrix did not show nonspecific interactions even in the presence of complex biological samples such as sera. Further work is currently ongoing in our laboratories to consolidate and expand these results and to apply this strategy in highly challenging assays.

## 4. Conclusions and Perspectives

The use of peptide-based materials for diagnostic purposes holds a great deal of potential for further development and applications. It is tempting to suggest that methods for the de novo prediction of interacting sequences can be used not only to mimic antigen–antibody interactions but complex (even multivalent) interactions in general [58,59,60,61,62]. The latter are an extremely challenging class of targets, due to the peculiar conformational and chemical characteristics of the interacting surfaces; the selection, design, and optimal display of mimics of the regions that underpin protein–protein molecular recognition can be aptly used to detect specific PPIs in normal vs. transformed cells. In this context, polyvalent interactions, connected to the presentation of multiple binding sequences, can collectively yield much richer information and stronger responses than their single-sequence counterparts, generating new opportunities for diagnosis as well as details on the basis for mechanisms which are apt to agonize or antagonize biological interactions. 

In this context, the improvement in multiscale methods will permit us to simulate events such as the recognition of multiple receptors on host-cells, virus entry or bacterial invasion with a level of detail that can be directly translated into new design rules for the development of biomimetic systems [63,64,65]. Moreover, the dramatic expansion of computer learning methods and the ever-increasing computational power will facilitate the application of artificial intelligence approaches aimed at exploiting the wealth of data made available by genomics, proteomics, epidemiology programs (to name a few) into generating new methods for the design and engineering of peptide-based probes. Machine learning algorithms, for instance, could be used to reveal non-trivial patterns that exist between specific recognition sequences, their functions in their cognate proteins and biochemical processes, and their reverberations at the systems level. Approaches of this kind have already been applied in protein secondary structure prediction, drug discovery, and the deimmunization of immunoreactive sequences [66,67,68,69,70,71,72].
